# Development and validation of a clinical model to predict low-grade intraepithelial neoplasia in chronic atrophic gastritis patients: a retrospective observational multicenter analysis

**DOI:** 10.3389/fonc.2025.1597099

**Published:** 2025-06-04

**Authors:** Wenjing Ding, Cheng Zhang, Hui Chen, Meng Gao, Xiaolong Xu, Bei Pei, Yi Zhang, Biao Song, Xuejun Li

**Affiliations:** ^1^ The Second Clinical Medical School, Anhui University of Chinese Medicine, Hefei, China; ^2^ Department of Research, The Second Affiliated Hospital of Anhui University of Chinese Medicine, Hefei, China; ^3^ Department of Gastroenterology, Hefei Second People’s Hospital, Hefei, China; ^4^ Department of Gastroenterology, The Second Affiliated Hospital of Anhui University of Chinese Medicine, Hefei, China

**Keywords:** chronic atrophic gastritis, low-grade intraepithelial neoplasia, multi-center retrospective analysis, predictive model, nomogram

## Abstract

**Background:**

Chronic atrophic gastritis (CAG), an early stage of gastric cancer, is a major digestive disorder, and the prognosis of CAG is determined by many sociodemographic and clinicopathologic subject characteristics. This retrospective observational multicenter analysis was conducted to explore risk factors and construct a predictive model for low-grade intraepithelial neoplasia (LGIN) in patients with CAG.

**Methods:**

The training dataset included 317 CAG patients diagnosed and treated in the Second Affiliated Hospital of Anhui University of Chinese Medicine from September 2018 to January 2025. All the baseline characteristics, including gender, age, education, basic diseases, blood indicators, and pathological mechanism during treatment of CAG, were recorded and selected based on both the least absolute shrinkage and selection operator (LASSO) regression analysis with 10-fold cross-validation and logistic regression analysis. After that, the nomogram was established, and its accuracy and predictive performance were evaluated via the area under the receiver operating characteristic (ROC) curves (AUC), calibration curves, Hosmer–Lemeshow goodness-of-fit test, and decision curve analysis (DCA) curves. For the validation dataset, the medical record information of 92 CAG patients diagnosed and treated in the Hefei Second People’s Hospital from November 2023 to January 2025 was recorded for subsequent analysis.

**Results:**

Our LASSO regression analysis revealed that family history, HP infection, pepsinogen I, pepsinogen II, bile reflux, and Kimura–Takemoto classification (C3 vs. C1) were significant independent risk factors, and the fitting equation was obtained. A nomogram for predicting LGIN in CAG patients was established. The ROC curve revealed that our predictive model showed good predictive efficacy with an AUC value of 0.838 (95% CI = 0.789–0.887) with a specificity of 0.761 and a sensitivity of 0.791 in the training dataset and an AUC value of 0.941 (95% CI = 0.893–0.989) with a specificity of 0.852 and a sensitivity of 0.908 in the validation dataset. Moreover, calibration and DCA curves demonstrated that our predictive model had a good fit, better net benefit, and predictive efficiency in LGIN in CAG patients.

**Conclusions:**

Our predictive model demonstrated that family history, HP infection, pepsinogen I, pepsinogen II, bile reflux, and Kimura–Takemoto classification were the independent risk factors of LGIN in CAG patients with high accuracy and good calibration.

## Introduction

Chronic atrophic gastritis (CAG) is a progressive inflammatory condition characterized by the thinning and degradation of the gastric mucosa, accompanied by the loss of functional gastric glands (1, 2), and it remains a significant global health concern, particularly affecting aging populations and regions with high *Helicobacter pylori* (HP) infection rates, such as parts of East Asia, Eastern Europe, and South America (3). Symptoms of CAG are diverse and may include abdominal discomfort, often presenting as a dull pain, fullness, or a burning sensation in the upper abdomen. Nausea and vomiting are also prevalent, especially after meals. Some patients may experience a reduced appetite, leading to weight loss over time (4). Meanwhile, CAG may progress to more severe conditions, and the risk of developing peptic ulcers increases as the gastric mucosa deteriorates (5). Moreover, long-term inflammation can cause intestinal metaplasia, a precancerous change that significantly raises the risk of gastric adenocarcinoma (6, 7).

The cause of CAG is often derived from long-term HP infection (8), autoimmune responses (9), or chronic exposure to persistent irritations such as alcohol consumption, smoking, long-term bile reflux, or intake of non-steroidal anti-inflammatory drugs (NSAIDs) (10). Over time, the damaged mucosa may undergo intestinal metaplasia, where stomach cells are replaced by intestinal-type cells, impairing acid production and digestive function (11). Symptoms can range from mild indigestion and bloating to severe deficiencies in vitamin B_12_ or iron due to malabsorption (12). The progression of CAG to more severe precancerous stages, such as low-grade intraepithelial neoplasia (LGIN), is influenced by multiple factors encompassing HP and alcohol and tobacco intake. It is marked by cellular atypia, and architectural distortion confined to the epithelial layer often emerges in this milieu of chronic injury (13). Early detection through endoscopic surveillance and eradication of HP are critical to halting progression to high-grade dysplasia or invasive carcinoma (14). However, the diagnostic result of LGIN in CAG patients is still unclear.

Hence, this study was conducted to develop and validate a novel nomogram that incorporates clinicopathologic factors associated with LGIN based on a model for predicting LGIN in CAG patients.

## Materials and methods

### Data source and participants’ information

Our multicenter retrospective observational study was approved by the Ethics Committee of the Second Affiliated Hospital of Anhui University of Chinese Medicine and the Hefei Second People’s Hospital and followed the Declaration of Helsinki, which was a multiple-center, retrospective, and observational analysis on CAG patients admitted to two hospitals. The clinical data of 317 patients from September 2018 to January 2025 in the Department of Gastroenterology of the Second Affiliated Hospital of Anhui University of Chinese Medicine were utilized for our predictive model as the training dataset, and the clinical data of 92 patients from November 2023 to January 2025 in the Department of Gastroenterology of the Hefei Second People’s Hospital were utilized for our predictive model as the validation dataset.

The inclusion criteria for our clinical data collection were as follows: a) age over 18 years old, b) patients diagnosed through endoscopy or pathology demonstrating CAG, c) patients having complete and searchable clinical information such as blood biomarkers and CAG classification data, and d) patients participating in our retrospective observational study voluntarily. The exclusion criteria were a) patients with CAG who received medical treatment in the past, b) patients having incomplete clinical data, and c) patients who were not willing to participate in our retrospective observational study.

The diagnosis of gastric LGIN requires comprehensive evaluation through multimodal methods. First, endoscopic examination is the core means. Conventional gastroscopy can initially identify mucosal abnormalities (such as erythema, erosion, or mucosal roughness), while enhanced imaging techniques (such as narrow band imaging, magnifying endoscopy, or chromoendoscopy) can further observe microvascular and glandular structural changes and assist in locating suspicious areas. Secondly, histopathological analysis is the key to diagnosis, and multiple biopsies (at least three to five pieces) are required to cover the range of lesions to avoid missed diagnosis; microscopic features include mild nuclear atypia and disordered arrangement, but the lesions are limited to the lower half of the mucosa and need to be differentiated from inflammation or reparative hyperplasia. In addition, HP detection is indispensable because HP infection is an important cause of LGIN, and eradication therapy may reverse some lesions. Finally, it is necessary to combine clinical follow-up to dynamically evaluate the condition. Some cases may progress to high-grade lesions or cancer, and timely intervention is required. During the diagnostic process, attention should be paid to the consistency differences between pathologists, and multidisciplinary consultation is recommended to improve accuracy. In general, the diagnosis of LGIN relies on the close integration of endoscopy and pathology, combined with etiological evaluation and dynamic monitoring, to provide a basis for subsequent treatment decisions.

In our retrospective analysis, the least absolute shrinkage and selection operator (LASSO) and multivariate logistic regression were utilized to establish our predictive model. In order to prevent overfitting of our predictive model, the number ratio (317:92) between the training dataset and the validation dataset, approximately 7:3 or 8:2, is reasonable (15).

### Characteristic selection

Similar to the aforementioned literature (16, 17) and the aim of our retrospective analysis, the following characteristics were analyzed and studied: a) sociodemographic characteristics [gender of patients (male or female), age at diagnosis, education (less than primary school, middle school, or upper college), and marital status (single or married)], b) questionnaire information [obesity (no, yes), hypertension (no, yes) (systolic blood pressure 140 or diastolic blood pressure 90), depression (no, yes), frailty (no, yes), alcohol consumption (no, yes), smoking (no, yes), diabetes (no, yes), family history (no, yes), dyslipidemia (no, yes) (cholesterol 6.19 or low-density lipoprotein, LDL 4.14)], c) laboratory data [HP infection (no, yes), glucose (mmol/L), cholesterol (mmol/L), LDL (mmol/L), pepsinogen I (μg/L), pepsinogen II (μg/L), gastrin 17 (pmol/L), alpha fetoprotein (AFP) (μg/L), carcinoembryonic antigen (CEA) (μg/L), carbohydrate antigen 125 (CA125) (U/mL), carbohydrate antigen 199 (CA199) (U/mL), and D-dimer (mg/L)], and d) endoscopic characteristics [Kimura–Takemoto (KT) classification (C1, C2 or C3) and bile reflux (no, yes)].

### Statistical analysis

Data were analyzed using R software 4.4.2 in our retrospective analysis. Continuous characteristics were presented as median (interquartile range), *p*-values were calculated via the Mann–Whitney *U* test, and comparisons between groups were analyzed by the rank sum test. Categorical characteristics were presented as number (*N*) or proportion (%), and comparisons between groups were analyzed using the chi-squared test or Fisher’s exact test. We employed the correlative analysis of independent characteristics via the function “cor()” and a heatmap of a correlation matrix was obtained, showing a graphical representation that utilized color-coding to visualize the strength and direction of the relationships between characteristics in a dataset. Each cell in the matrix showed the correlation coefficient ranging from −1 to 1 based on Pearson’s method. Furthermore, a nomogram was plotted to illustrate the risk of LGIN in patients with CAG, and the LASSO regression analysis was utilized to select relevant characteristics to establish our predictive model of high-dimensional data (18). After selecting predictors of LGIN in patients with CAG, the 10-fold cross-validation based on the LASSO regression analysis was utilized to confirm the suitable tuning parameters (*λ*), and the coefficients of a sparse matrix with non-zero as selected characteristics via the minimum *λ* were considered (19). Selected characteristics were applied via the multivariate logistic regression analysis, and *p*-values less than 0.05 were considered as independent variables of the radiomics nomogram (20).

The performance of our predictive model was assessed in both the training and validation datasets, including the assessment of the receiver operating characteristic (ROC) curve (AUC), sensitivity, and specificity. Moreover, the calibration curve and Hosmer–Lemeshow goodness of fit were used to evaluate the effectiveness (21). Decision curve analysis (DCA) was conducted to validate the accuracy of the predictive model by quantifying the net benefits at different threshold probabilities (22).

In the construction of the clinical model and in the subsequent analysis, R version 4.4.2 (http://www.r-project.org, R Foundation for Statistical Computing) was utilized. For baseline characteristics, we utilized the “tableone” package, and the table was drawn via both the “flextable” and “officer” packages. In the LASSO regression analysis, the “glmnet” R package was utilized. For establishing the linear regression model, the “rms” package was used to plot the radiomics nomogram for subsequent analysis. We plotted the ROC and DCA curves based on the “pROC” and “rmda” packages, respectively, and the R package “ResourceSelection” was used to perform the Hosmer–Lemeshow goodness of fit. A two-bilateral *p*-value less than 0.05 was considered statistically significant.

## Results

### Participant baseline characteristics

Detailed baseline information of the sociodemographic and clinicopathologic characteristics of both the training and validation datasets is shown in **Table 1**. For the training dataset, with a total of 317 CAG patients with non-LGIN (*N* = 92, 29%) and LGIN (*N* = 225, 71%), consisting of 144 male patients (45.4%) and 173 female patients (54.6%), the mean (SD) age was 62.03 ± 12.11 in non-LGIN patients and 61.28 ± 11.91 in LGIN utilized. For the validation dataset, with a total of 92 CAG patients with non-

**Table 1:** Baseline characteristics based on CAG patients with non-LGIN and LGIN.

Characteristics	Training dataset	Validation dataset	*p*-value
	(*N* = 317)	(*N* = 92)	
Gender			0.692
Male	144 (45.4)	39 (42.4)	
Female	173 (54.6)	53 (57.6)	
Age, mean (SD)	61.50 (11.95)	60.09 (13.18)	0.332
Education			0.458
Less than primary school	128 (40.4)	33 (35.9)	
Middle school	100 (31.5)	27 (29.3)	
Upper college	89 (28.1)	32 (34.8)	
Marital status			0.478
Married	307 (96.8)	87 (94.6)	
Single	10 (3.2)	5 (5.4)	
Obesity			0.697
Yes	130 (41.0)	35 (38.0)	
No	187 (59.0)	57 (62.0)	
Hypertension			<0.001
Yes	148 (46.7)	72 (78.3)	
No	169 (53.3)	20 (21.7)	
Depression			0.229
Yes	268 (84.5)	83 (90.2)	
No	49 (15.5)	9 (9.8)	
Frailty			0.387
Yes	269 (84.9)	82 (89.1)	
No	48 (15.1)	10 (10.9)	
Alcohol consumption			0.127
Yes	243 (76.7)	78 (84.8)	
No	74 (23.3)	14 (15.2)	
Smoking			0.250
Yes	223 (70.3)	71 (77.2)	
No	94 (29.7)	21 (22.8)	
Diabetes			0.105
Yes	95 (30.0)	19 (20.7)	
No	222 (70.0)	73 (79.3)	
Dyslipidemia			<0.001
Yes	95 (30.0)	19 (20.7)	
No	296 (93.4)	62 (67.4)	
Family history			0.097
Yes	243 (76.7)	62 (67.4)	
No	74 (23.3)	30 (32.6)	
HP infection			0.008
Yes	254 (80.1)	61 (66.3)	
No	63 (19.9)	31 (33.7)	
Glucose, mean (SD), mmol/L	5.14 (0.84)	5.50 (1.15)	0.001
Cholesterol, mean (SD), mmol/L	4.39 (1.02)	4.47 (1.67)	0.564
Pepsinogen I, mean (SD), μg/L	111.24 (26.86)	110.57 (26.92)	0.832
Pepsinogen II, mean (SD), μg/L	9.48 (3.43)	11.00 (3.62)	<0.001
Gastrin 17, mean (SD), pmol/L	7.50 (3.52)	7.64 (3.50)	0.734
AFP, mean (SD), μg/L	5.61 (4.97)	9.05 (5.31)	<0.001
CEA, mean (SD), μg/L	2.11 (1.12)	2.57 (1.47)	0.001
CA125, mean (SD), U/mL	9.82 (10.13)	16.81 (8.89)	<0.001
CA199, mean (SD), U/mL	15.66 (8.05)	18.77 (10.62)	0.003
D-dimer, mean (SD), mg/L	0.34 (0.13)	0.35 (0.15)	0.537
KT classification			0.417
C1	102 (32.2)	23 (25.0)	
C2	107 (33.8)	35 (38.0)	
C3	108 (34.1)	34 (37.0)	
Bile reflux			0.435
Yes	239 (75.4)	65 (70.7)	
No	78 (24.6)	27 (29.3)	

LGIN (*N* = 27, 29.3%) and LGIN (*N* = 65, 70.7%), consisting of 39 male patients (42.4%) and 53 female patients (57.6%), the mean (SD) age was 60.59 ± 15.36 in non-LGIN patients and 59.88 ± 12.30 in LGIN patients, as shown in **Supplementary Table S1** and **Table 1**. Among the characteristics in both the training and validation datasets, nine characteristics showed significant differences between the training and validation datasets: hypertension (*p* < 0.001), dyslipidemia (*p* < 0.001), HP infection (*p* = 0.008), glucose (*p* = 0.001), pepsinogen II (*p* < 0.001), AFP (*p* < 0.001), CEA (*p* = 0.001), CA125 (*p* < 0.001), and CA199 (*p* = 0.003).

### Correlation heatmap of the predictive characteristics in the training dataset

**Figure 1:**
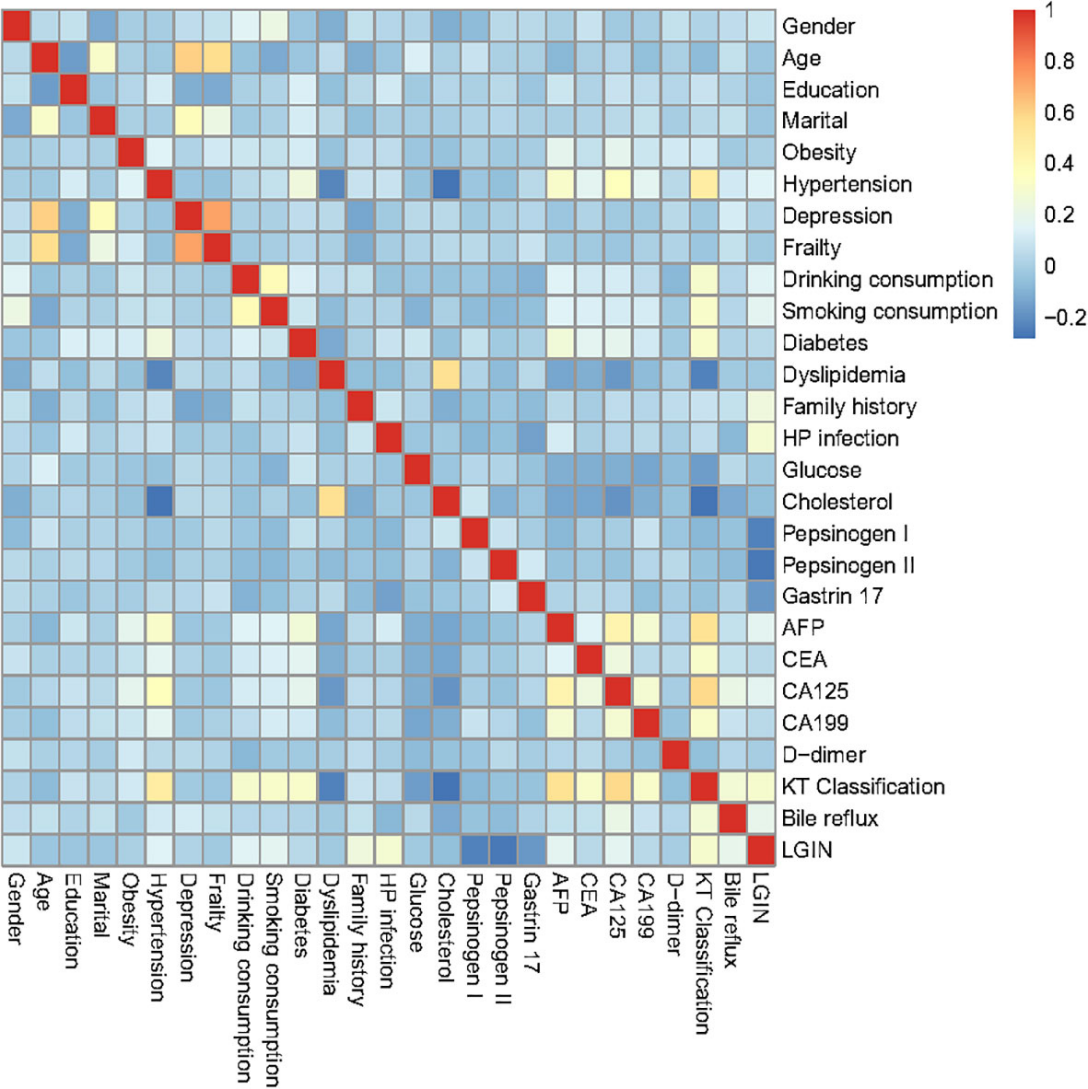
The heatmap of the correlation matrix of the characteristics in the training dataset.

As depicted in **Figure 1**, the correlation matrix heatmap of our training dataset revealed the causal associations between different predictive characteristics. In our heatmap of our correlation matrix, warm colors (e.g., red) often indicate strong positive correlations, cool colors (e.g., blue) represent negative correlations, and neutral colors (e.g., white) denote weak or no correlation. A correlation analysis was performed on the predictive variables of our model, which included gender, age, level of education, marital status, obesity, hypertension, depression, frailty, alcohol consumption, smoking, diabetes, dyslipidemia, family history, HP infection, glucose, cholesterol, pepsinogen I, pepsinogen II, gastrin 17, AFP, CEA, CA125, CA199, D-dimer, Kimura–Takemoto classification, and bile reflux. Considering that all characteristics have weak correlation and no multicollinearity in the visualization of the heatmap, we utilized these recorded characteristics for the subsequent LASSO regression analysis in our retrospective study.

### Selection of predictive characteristics and nomogram establishment

**Figure 2:**
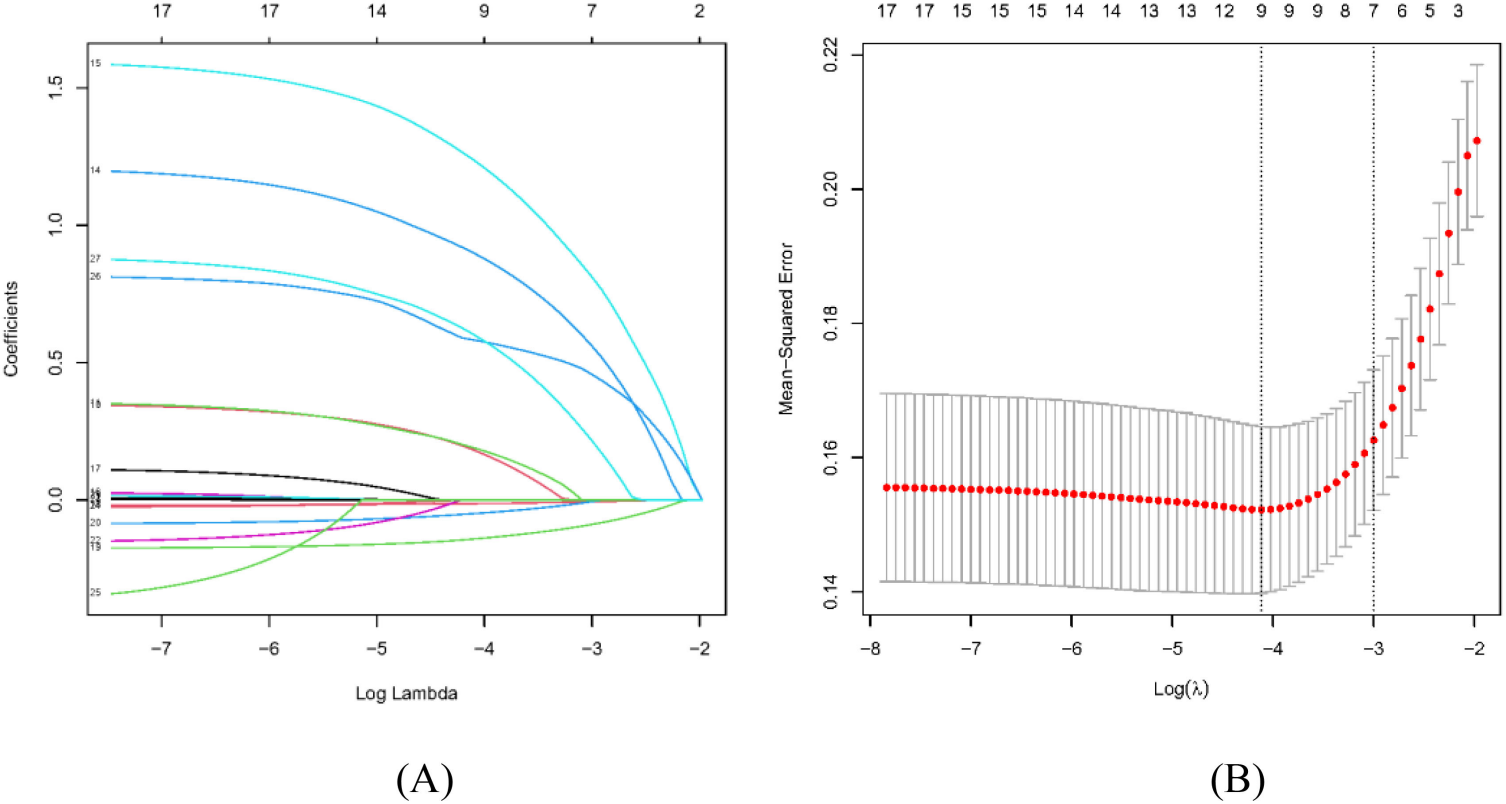
Selection of sociodemographic and clinicopathologic characteristics using the LASSO regression analysis. **(A)** The LASSO coefficient profiles of the 17 texture features. A coefficient profile plot was produced against the log(*λ*) sequence. By using 10-fold cross-validation, 24 non-zero coefficients based on optimal *λ* were selected. **(B)** The optimal parameter (*λ*) in the LASSO model was selected via 10-fold cross-validation using minimum criteria. The left dashed line represents *λ*.min and the right dashed line represents *λ*.1se.


In our LASSO logistic regression analysis, we leveraged 10-fold cross-validation to obtain the optimal parameter *λ* for our predictive model and finally screened nine characteristics: alcohol consumption, smoking, family history, HP infection, glucose, pepsinogen I,

pepsinogen II, gastrin 17, bile reflux, and Kimura–Takemoto classification (*p* < 0.05), as depicted in **Figure 2**. Two LASSO result figures have chosen non-zero coefficients as the underlying factors of LGIN. Based on the results of the LASSO logistic regression analysis, the fitting equation of our predictive model is as follows:

$$\text{LASSO}\left(\text{P}\right)=0.601+0.025x_{1}+0.037x_{2}+0.166x_{3}+0.234x_{4}-0.002x_{5}-$$

$$0.023x_{6}-0.008x_{7}+0.097x_{8}+0.107x_{9}$$

where $x_{1}$ represents alcohol consumption (No denotes 0, Yes denotes 1), $x_{2}$ represents smoking (No denotes 0, Yes denotes 1), $x_{3}$ represents family history (No denotes 0, Yes denotes 1), $x_{4}$ represents HP infection (No denotes 0, Yes denotes 1), $x_{5}$ represents pepsinogen I value, $x_{6}$ represents pepsinogen II value, $x_{7}$ represents gastrin 17 value, $x_{8}$ represents bile reflux (No denotes 0, Yes denotes 1), $x_{9}$ represents Kimura–Takemoto classification (C1, C2, C3), and the constant term of the formula (0.601) means reference intercept.

Multivariate logistic regression analysis further showed that family history (OR = 3.111, 95% CI = 1.620–6.039), HP infection (OR = 4.810, 95% CI = 2.335–10.16), pepsinogen I (OR = 0.982, 95% CI = 0.970–0.993), pepsinogen II (OR = 0.832, 95% CI = 0.755–0.912), bile reflux (OR = 2.388, 95% CI = 1.212–4.727), and Kimura–Takemoto classification (C3 vs. C1) (OR = 3.874, 95% CI = 1.693–9.264) are independent risk factors for LGIN in CAG patients (*p* < 0.05), respectively, as presented in **Table 2**. The *β* values of three characteristics, encompassing pepsinogen I, pepsinogen II, and gastrin 17, were all less than zero, and the OR values of the aforementioned characteristics were also less than zero, which means that these three characteristics are the protective factors for LGIN in CAG patients.

**Table 2:** Multivariate logistic analysis of relevant risk factors related to LGIN of CAG patients.

Characteristic	*β*	sx	Wald’s	OR (95% CI)	*p*-value
Alcohol consumption	0.382	0.374	1.043	1.465 (0.701–3.053)	0.307
Smoking	0.343	0.355	0.935	1.41 (0.699–2.826)	0.333
Family history	1.135	0.335	11.507	3.111 (1.62–6.039)	<0.001
HP infection	1.571	0.374	17.668	4.81 (2.335–10.16)	<0.001
Pepsinogen I	−0.018	0.006	9.568	0.982 (0.97–0.993)	0.002
Pepsinogen II	−0.184	0.048	14.522	0.832 (0.755–0.912)	<0.001
Gastrin 17	−0.081	0.045	3.338	0.922 (0.843–1.005)	0.068
Bile reflux	0.87	0.346	6.327	2.388 (1.212–4.727)	0.012
KT classification C2 vs. C1	0.533	0.379	1.976	1.703 (0.812–3.605)	0.160
KT classification C3 vs. C1	1.354	0.431	9.856	3.874 (1.693–9.264)	0.002

**Figure 3:**
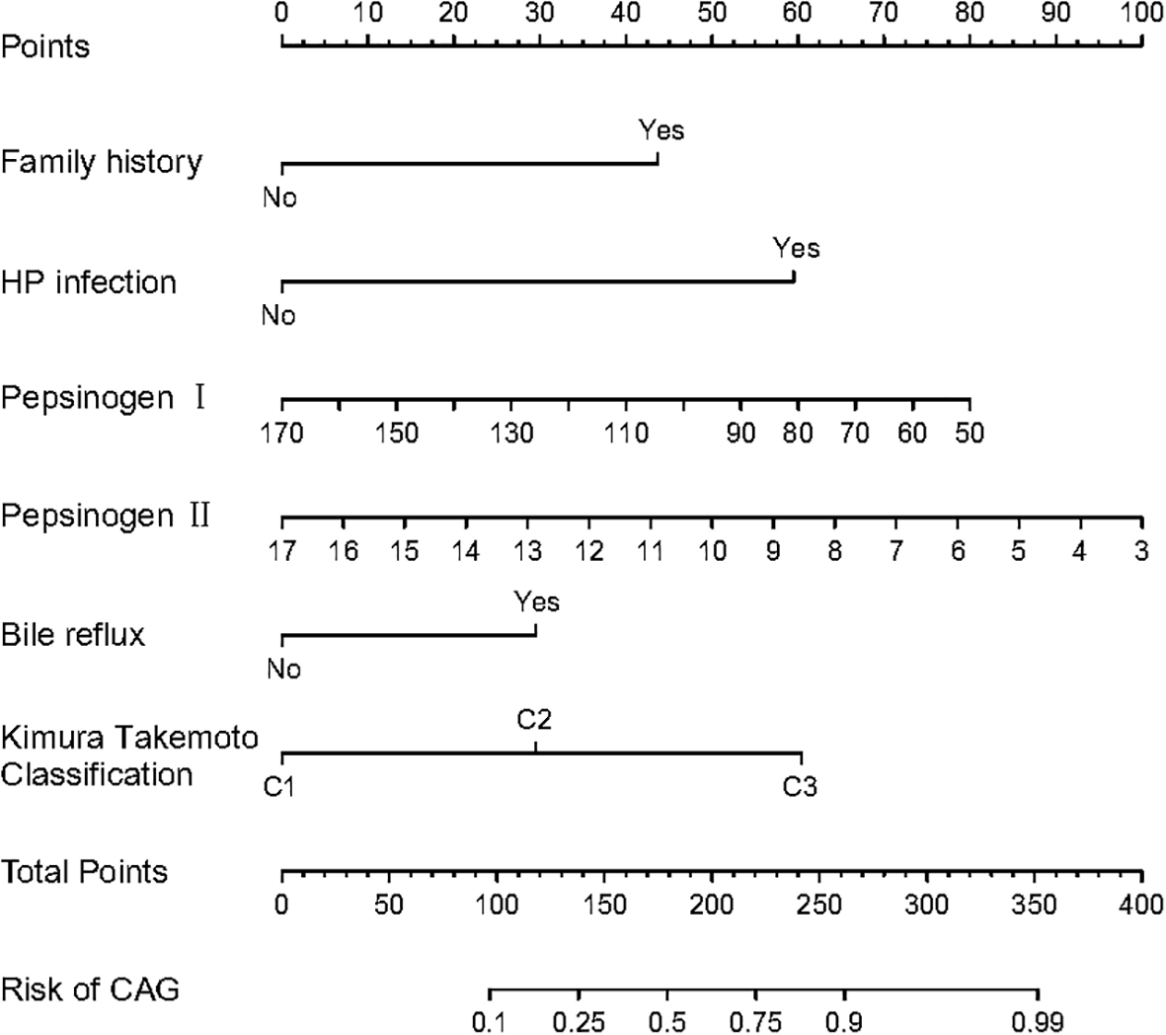
The nomogram for predicting LGIN in CAG patients.

Meanwhile, the predictive model was plotted as a radiomics nomogram, constructed using family history, HP infection, pepsinogen I, pepsinogen II, bile reflux, and Kimura–Takemoto classification based on the aforementioned risk factors, as shown in **Figure 3**. Our radiomics nomogram provides a visual representation of the impact of each factor, helping doctors in conducting individualized risk evaluations in clinical practice. For example, if a patient with CAG had family history, HP infection with pepsinogen I (170) and pepsinogen II (17), bile reflux, and C2 classification, then the patient’s corresponding scores would be approximately 43, 60, 0, 0, 30, and 30, respectively, with a total score of 133. This would reveal that the estimated probability of CAG patients with LGIN is approximately 23%.

### Validation and calibration of the predictive model

**Figure 4:**
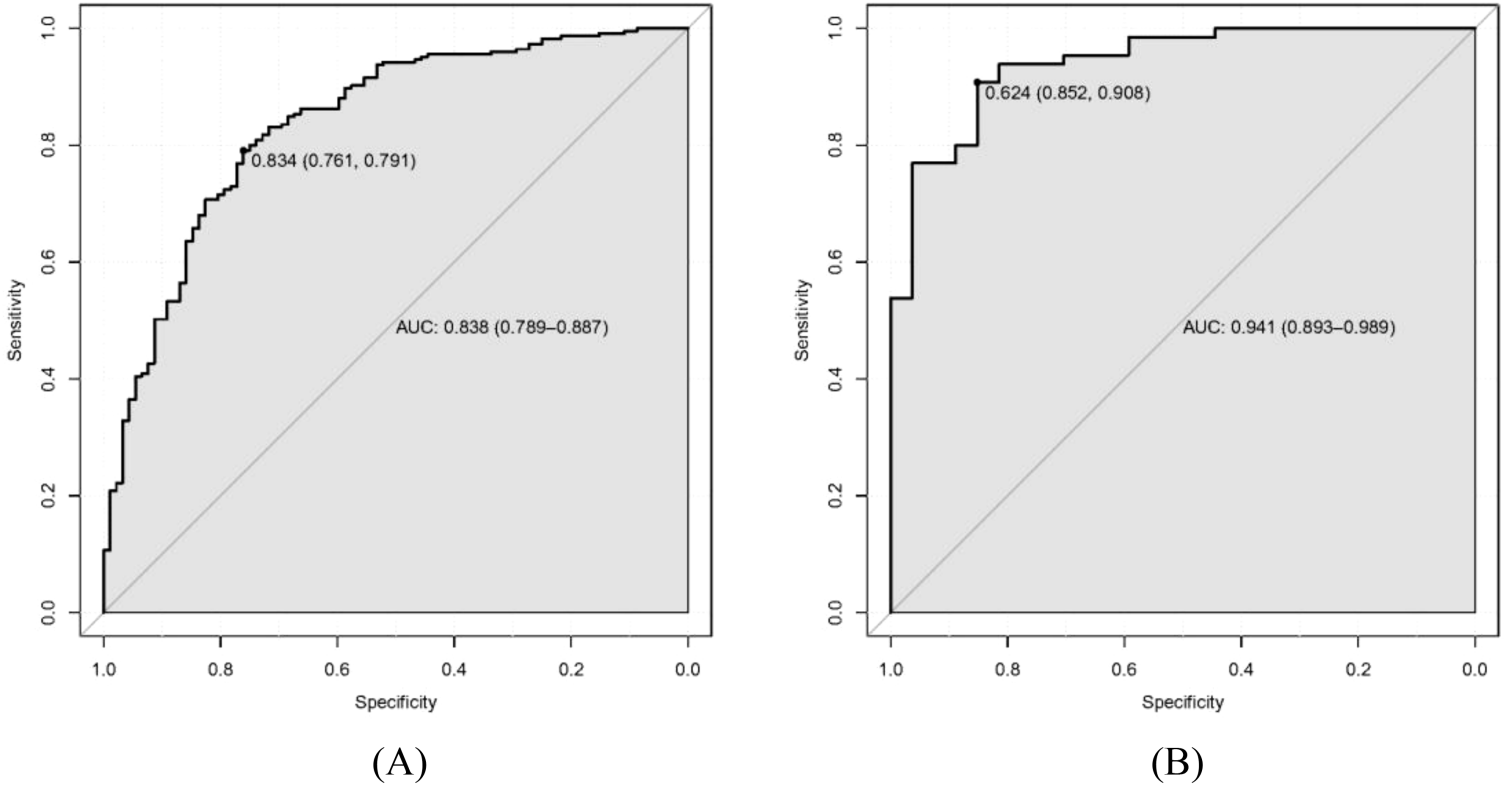
Receiver operating characteristic curve (ROC) of our predictive model. **(A)** Training dataset. **(B)** Validation dataset.

**Figure 5:**
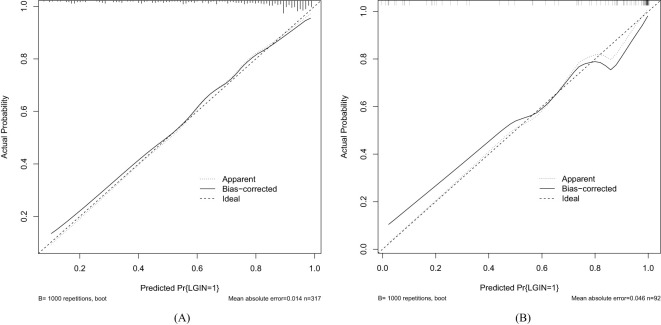
Calibration curve of our predictive model. **(A)** Training dataset. **(B)** Validation dataset.

**Figure 6:**
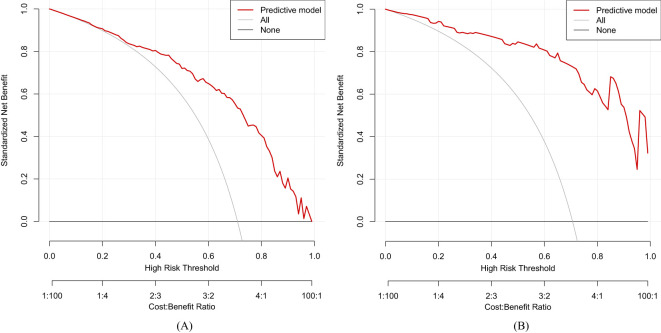
Decision curve analysis (DCA) of our predictive model. **(A)** Training dataset. **(B)** Validation dataset.

As shown in **Figures 4A, B**, the discrimination power of our predictive model was assessed by AUC

values calculated in ROC figures by analyzing the indication of LGIN in CAG patients in both the training and validation datasets. The ROC figure in the training dataset calculated an AUC value of 0.838 (95% CI = 0.789–0.887) with a specificity of 0.761 and a sensitivity of 0.791 as well as an AUC value of 0.941 (95% CI = 0.893–0.989) with a specificity of 0.852 and a sensitivity of 0.908 in the validation dataset. The AUC in the validation dataset (0.941) had a higher score than in the training dataset (0.838), which means that our predictive model has a good effect. Furthermore, the Hosmer and Lemeshow goodness-of-fit (GOF) test and the calibration curve were utilized to evaluate our predictive model, and a *p*-value of the Hosmer and Lemeshow GOF test greater than 0.05 indicates that the predictive model has a good degree of fit. The results showed that our model had a good fit for the training (*χ* ^2^ = 4.1407, *df* = 8, *p*-value = 0.8442) and validation datasets (*χ* ^2^ = 3.3873, *df* = 8, *p*-value = 0.9078). The calibration curves for the radiomics nomogram based on our multivariate logistic regression analysis in the training and validation datasets are depicted in **Figures 5A, B** and demonstrated good agreement between the prediction results and the observational outcomes. In clinical practice, calibration curves are commonly used to evaluate and optimize predictive models such as the probability of postoperative complications for LGIN in CAG patients. By comparing the predicted probability with the actual occurrence probability, doctors can develop more accurate postoperative monitoring and intervention plans. Moreover, for decision curve analysis, the DCA in both the training and validation datasets indicated that the net benefit of our predictive model was consistently better than the two extreme strategies (all treatment and no treatment) across a wide range of threshold probabilities, representing its underlying clinical ability, as shown in **Figure 6**. According to the DCA, doctors can choose the most appropriate intervention threshold based on changes in net benefit. This helps avoid excessive or inappropriate intervention and improve the quality of medical decision-making for LGIN in CAG patients. Good calibration curve and the DCA revealed that our model has good calibration, clinical application, and generalization.

## Discussion

Our multicenter retrospective analysis established a clinical predictive model based on LASSO and multivariate logistic regression algorithm. For baseline results, nine characteristics, namely, hypertension (*p* < 0.001), dyslipidemia (*p* < 0.001), HP infection (*p* = 0.008), glucose (*p* = 0.001), pepsinogen II (*p* < 0.001), AFP (*p* < 0.001), CEA (*p* = 0.001), CA125 (*p* < 0.001), and CA199 (*p* = 0.003), showed significant differences. The LASSO and multivariate logistic regression method evaluated the influence of family history, HP infection, pepsinogen I, pepsinogen II, bile reflux, and Kimura–Takemoto classification on the impact of LGIN in CAG patients. Some literature suggested that LGIN may act as a critical transitional stage in the progression of CAG to advanced premalignant lesions, with its presence correlating with more severe mucosal atrophy and intestinal metaplasia (23–25). Notably, CAG patients with LGIN exhibited a higher likelihood of multifocal atrophy and accelerated histological deterioration, aligning with prior evidence that LGIN serves as a marker of genomic instability in the gastric mucosa (26). Our retrospective observational analysis aimed to evaluate the diagnostic efficacy of LGIN in combination with other characteristics for the concurrent detection of CAG, in order to offer a basic foundation for the diagnosis and treatment of CAG disease.

A family history of gastric cancer or premalignant conditions was significantly associated with advanced CAG, with clinical and molecular evidence highlighting inherited susceptibility as a key modifier of disease severity. Epidemiological studies consistently demonstrate that individuals with a family history of gastric cancer or premalignant gastric lesions exhibit a two- or three-fold increased risk of developing advanced CAG, independent of HP infection or environmental exposures (27, 28). This association is likely mediated by genetic polymorphisms in pathways regulating gastric mucosal homeostasis, such as pro-inflammatory cytokines (29), tumor suppressor genes (30), and genes involved in acid secretion (31). Clinically, patients with a family history often present with earlier-onset, multifocal atrophy and accelerated progression to intestinal metaplasia or dysplasia (32).

HP infection is a major etiological factor in the development of CAG, a condition characterized by progressive loss of gastric glandular structures and mucosal thinning (33). HP colonizes the gastric epithelium, triggering a persistent inflammatory response mediated by bacterial virulence factors (e.g., CagA and VacA toxins) and host immune reactions (34). Over time, chronic inflammation disrupts gastric homeostasis, leading to glandular atrophy, parietal cell loss, and hypochlorhydria (reduced stomach acid secretion) (35). These pathological changes are hallmarks of CAG and significantly increase the risk of metaplastic transformations, such as intestinal metaplasia, which is a precursor to gastric cancer (36). Several studies revealed that long-term HP infection accelerates the progression from non-atrophic gastritis to CAG, with bacterial persistence, host genetic susceptibility, and environmental cofactors (e.g., smoking, high-salt diet) influencing disease severity (37, 38).

In CAG patients, progressive atrophy of the gastric glands leads to reduced secretion of pepsinogen I (produced primarily in the gastric corpus/fundus), while pepsinogen II (produced throughout the stomach) levels remain relatively stable, and precursors of the digestive enzyme pepsin serve as important biomarkers for CAG (39). This results in a characteristic decrease in the pepsinogen I/pepsinogen II ratio, which has become a validated non-invasive diagnostic indicator for gastric mucosal atrophy (40). Serum pepsinogen testing (pepsinogen I 70 μg/L and pepsinogen I/pepsinogen II ratio 3) is widely used to screen for CAG, particularly in high-risk populations for gastric cancer (41). The severity of corpus atrophy correlates strongly with declining pepsinogen I levels, reflecting the loss of acid-secreting parietal cells and enzyme-producing chief cells in CAG progression. This serological approach is especially valuable for detecting early-stage atrophy before endoscopically visible changes occur (42).

Bile reflux, the backward flow of duodenal contents (including bile acids, pancreatic enzymes, and intestinal fluid) into the stomach, is increasingly recognized as a contributing factor to CAG. Prolonged bile reflux damages the gastric mucosal barrier through multiple mechanisms: bile acids disrupt surface mucous cells, induce oxidative stress, and trigger chronic inflammation, accelerating glandular atrophy (43, 44). This process often coexists with HP infection, creating a synergistic effect that exacerbates mucosal injury and impairs healing (45). Bile acids also inhibit proton pump function, reducing gastric acid secretion and altering the gastric microenvironment, which may further promote epithelial metaplasia and atrophy (46). Endoscopically, bile reflux is associated with mucosal erythema, erosions, and bile-stained fluid in the stomach. Chronic exposure to bile reflux correlates with advanced CAG stages and intestinal metaplasia, raising the risk of gastric carcinogenesis (47).

The Kimura–Takemoto classification is a widely used endoscopic grading system that evaluates the extent and pattern of gastric mucosal atrophy in CAG (48). It categorizes atrophy into two main types: closed type (C-type) and open type (O-type), based on the progression of atrophic borders observed during endoscopy. In closed-type atrophy, the atrophic changes remain confined to the lesser curvature of the stomach, while open-type atrophy involves expansion toward the greater curvature and fundus, reflecting more advanced disease (49). In our Second Affiliated Hospital of Anhui University of Chinese Medicine, the Hefei Second People’s Hospital, and our baseline of retrospective analysis, we only recorded the C-type in CAG patients. This classification correlates closely with histopathological severity, acid secretion levels, and gastric cancer risk (50). The Kimura–Takemoto system aids clinicians in stratifying CAG patients for surveillance, as open-type patterns warrant closer endoscopic monitoring due to their strong link to gastric carcinogenesis (51).

LGIN represents a precancerous lesion in the stomach and is closely associated with CAG. In CAG, prolonged mucosal inflammation and glandular atrophy create a microenvironment conducive to genetic and epigenetic alterations, promoting the development of cellular dysplasia (52). LGIN, characterized by mild-to-moderate architectural distortion and cytological atypia confined to the epithelial layer, frequently arises in areas of CAG with intestinal metaplasia (53). LGIN represents an early neoplastic transformation within this spectrum, marked by architectural distortion and cytological atypia confined to the epithelial layer (54). While LGIN itself carries a lower risk of progression to invasive adenocarcinoma compared to high-grade dysplasia, its presence in CAG significantly elevates cancer risk (55). The combination of atrophic changes, metaplasia, and dysplasia in CAG exemplifies the multistep “Correa cascade” of gastric cancer development (56).

Our retrospective observational analysis has several limitations. First, our study has selection bias. As a multicenter study, the cohort may not represent broader demographic or geographic populations, and the sample size of the validation cohort (*N* = 92) is not enough, and significant differences in some baseline characteristics, including hypertension, dyslipidemia, and HP infection, between the training and validation cohorts (as shown in **Table 1**) suggest intercenter variability, limiting model generalizability. Second, the LASSO and multivariate regression leveraged in our analysis are prevailing methods. Furthermore, machine learning algorithms can be utilized to establish a novel predictive model with better performance (57). Finally, there may be a bias in the record of more effective characteristics for our predictive model, encompassing short follow-up duration and serological and histological variability—the ratio of pepsinogen I/pepsinogen II and histopathological grading were subject to interlaboratory variability and interobserver discrepancies (58).

## Conclusion

In our multicenter retrospective analysis, we found a causal association between several independent factors (family history, HP infection, pepsinogen I, pepsinogen II, bile reflux, and Kimura–Takemoto classification) and LGIN in CAG patients and established a predictive model to evaluate the clinical diagnosis of CAG.

## Data Availability

Publicly available datasets were analyzed in this study. All recorded dataset can be obtained via email from the corresponding author.
